# High-order mode suppression in double-clad optical fibers by adding absorbing inclusions

**DOI:** 10.1038/s41598-020-63969-7

**Published:** 2020-04-28

**Authors:** Svetlana S. Aleshkina, Tatiana A. Kochergina, Vladimir V. Velmiskin, Konstantin K. Bobkov, Mikhail M. Bubnov, Mikhail V. Yashkov, Denis S. Lipatov, Mikhail Yu. Salganskii, Alexey N. Guryanov, Mikhail E. Likhachev

**Affiliations:** 1 Prokhorov General Physics Institute of the Russian Academy of Sciences, Dianov Fiber Optics Research Center, Moscow, 119333 Russia; 2 G.G. Devyatykh Institute of Chemistry of High-Purity Substances of the Russian Academy of Sciences, Nizhny Novgorod, 603950 Russia

**Keywords:** Fibre optics and optical communications, Design, synthesis and processing

## Abstract

We proposed and experimentally demonstrated a technique for the suppression of unwanted modes in double-clad fibers with a high core-to-clad diameter ratio by introducing high-index absorbing inclusions into the first cladding of the fibers. These inclusions disturb the shape of undesirable modes, and a noticeable part of the power becomes localized inside the inclusion, resulting in an increase in the propagation loss of these modes. Two fiber designs were studied and realized: one with cylindrical symmetry and an absorbing high-index ring as the inclusion and another with high-index absorbing rods inserted around the fiber core. In both cases, the possibility of achieving perfect single-mode propagation was demonstrated both theoretically and experimentally.

## Introduction

The invention of double-clad (DC) active optical fiber initiated the rapid development of high-power fiber laser systems. The ability to achieve diffraction-limited output beam quality and simultaneously inject a high pump power into the first fiber cladding led to the demonstration of single-mode fiber lasers with average powers up to 10 kW^1^. By now to achieve high peak power on the output of fiber laser systems novel coiling methods of the large-mode-area (LMA) fibers^2^ and novel double-clad fiber designs using, e. g., leakage-channel fibers^3,4^, tapered fibers^5,6^, by modal filtering through inserting small cores around the central core, which are resonant to the HOMs^7^ or by twisting the double-clad fiber with broken circular symmetry of the core^8^ have been proposed. Progress in the development of large-mode-area (LMA) fiber allowed further scaling of the peak power to the MW level directly after optical fiber amplifiers and to the GW level after compression of nanosecond pulses to femtosecond durations^9^.

At the same time, DC fibers are not truly single-mode in the conventional meaning. High-order modes under the cut-off wavelength in single-clad fiber propagate without any losses when a secondary reflection cladding is added. In the most common case, the core diameter D_core_ is an order of magnitude smaller than the first cladding diameter D_clad_, so the major part of the power in the high-order modes (HOMs) is localized in the first cladding, and only a small part is still confined in the fiber core. For this reason, these modes have negligible gain in fiber amplifiers and are not excited during fusion splicing of DC fiber with conventional single-mode fiber.

However, there are a number of applications in which active fiber with a large core-to-cladding diameter ratio is required. For example, efficient single-mode Yb-doped fiber lasers emitting near 0.98 μm^10–13^ and cladding-pumped single-mode Er-doped (Yb-free) fiber lasers and amplifiers have been developed^14,15^. Substantial progress in the development of LMA optical fiber completely changed the situation with “single-mode” DC fiber. When the “single-mode” core diameter becomes only a few times smaller than the first cladding diameter, the overlap integral between the cladding modes and the active core becomes considerable, and the cladding mode gain cannot be neglected anymore. For example, in^9^, the authors investigated photonic crystal fiber with a core diameter of 100 μm and a first cladding diameter of 340 μm and revealed that the cladding mode becomes efficiently amplified when the output power reaches 15 W.

In the present work, a new approach to achieve single-mode propagation in DC fiber with a large core-to-cladding diameter ratio is proposed and investigated. We add (first numerically, then we produce fiber preforms) high-index absorbing inclusions into the first reflecting cladding of fiber to disturb the HOM intensity distribution. By the other words, we propose the technique of undesirable modes suppression based on the delocalization of HOMs intensity from the fiber core and simultaneous its absorption in high index inclusions incorporated in the silica cladding. Two alternative optical fiber designs are considered: the first design has a step-index (SI) core surrounded by a high-index ring layer, and the second design has a SI core surrounded by a few high-index rods. By optimizing the inclusion parameters, we succeed in increasing the propagation losses of the HOMs and simultaneously keep a reasonably low optical loss for the fundamental mode.

It is worth noting that both designs require a very high loss level inside the inclusions at the signal wavelength. At the same time, active DC fibers are typically intended for cladding pumps and should have reasonably low loss at the pump wavelength. To satisfy both requirements, we propose utilization of absorbing elements doped with rare-earth ions. In this case, absorption on the order of hundreds (and even thousands) dB/m at the signal wavelength is possible when the pump wavelength loss might be as low as tens of dB/km^16^.

## Fiber with a high-index ring layer

### Structure design

To calculate the mode shape distributions in the case of the cylindrically symmetric fiber design, we used both in-house built software and the COMSOL software package. The fiber was optimized for operation in the spectral range of Er-doped fiber lasers (~1.55 μm) pumped into the first cladding at a wavelength near 0.98 μm. We considered configurations of DC fiber with a second cladding numerical aperture (NA) of 0.45. The designed fiber consisted of a SI core, which was surrounded by a depressed layer to reduce the HOM cut-off wavelength and make the fiber less sensitive to bending^17,18^. In previous works, utilization of such a refractive index profile (RIP) allowed the creation of single-mode fiber with a core diameter of 30–35 µm^14,15,19^.

**Figure 1:**
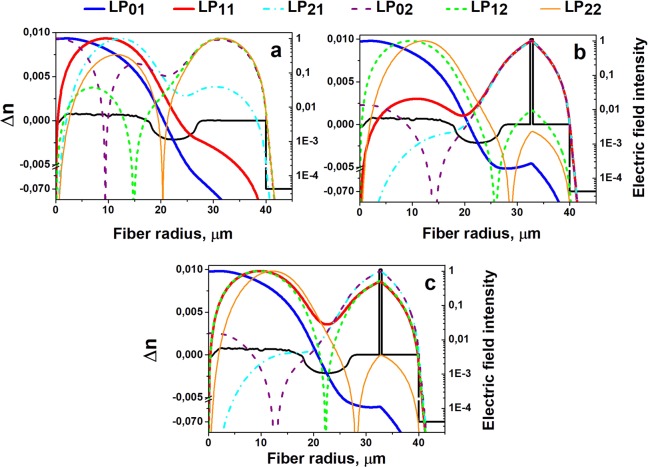
Calculated mode field intensity distribution for the DC-fiber design: (**a**) without an absorbing layer, (**b**) with an absorbing layer(thickness of 0.53 μm), (**c**) with the absorbing layer thickness “resonant” for coupling between the LP_11_ and LP_12_ modes (0.446 μm).

The RIP chosen for modeling is shown in Fig. 1. It has core and cladding diameters of 35 µm and 80 µm, respectively, and the core and cladding refractive index difference Δn_core_ is equal to 0.0007. Outside the core, a low-index layer with a refractive index below that of silica by 0.002 was introduced to reduce the HOM cut-off wavelength. The RIP was based on a measurement from a real preform, which we have in stock. In the case of an infinite silica cladding, the core was perfectly single-mode at an operating wavelength of 1.55 μm (the LP_11_ mode cut-off wavelength was 1.45 μm). As shown in Fig. 1a, adding the secondary reflecting cladding significantly changed the mode composition of the core. The fractional powers of the LP_11_ and LP_21_ modes in the core reach 94% and 84%, respectively, which in the case of active fiber design means the possibility of effective amplification of these modes.

The insertion of the high-index ring layer into the first fiber cladding allowed us to significantly change the electric field intensity distribution of the HOMs (Fig. 1b). Appropriate selection of the ring layer thickness resulted in most of the power in the LP_11_ and LP_21_ modes being localized outside the core (the electrical field intensity distributions of these modes have maxima near position of the absorption ring). Notably, instead of the LP_11_ mode, another HOM, LP_12_, becomes localized inside the core, so a more careful choice of layer thickness is required.

It should be noted that in general, the mode shape redistribution might not be sufficient to noticeably reduce undesirable mode gain in the case of active fibers and avoid excitation in the case of passive fibers. For this reason, our idea was to increase undesirable mode losses by making the inserted layer highly absorbing. In this case, even a small percentage of the total power located in the absorbing ring layer can lead to efficient HOM suppression. If we consider cladding pumped Er-doped (Yb-free) fibers the pump and signal wavelength are 0.98 µm and 1.55 μm correspondingly. The most efficient rare-earth absorbing dopant in our case is Tm^3+^, which has strong attenuation near 1.55 μm and low loss near 0.98 μm^16^. Its introduction into the silica glass network requires additional co-doping with P_2_O_5_ or Al_2_O_3_ to increase its solubility. This leads to the growth of the absorbing layer refractive index. Taking into account the technological requirements of simplicity and reliability in the fiber preform production, here we consider a fiber design with a refractive index difference between the absorbing layer and pure silica cladding of Δn_layer_ = 0.005–0.020 (in Fig. 1b Δn_layer_ = 0.010).

**Figure 2:**
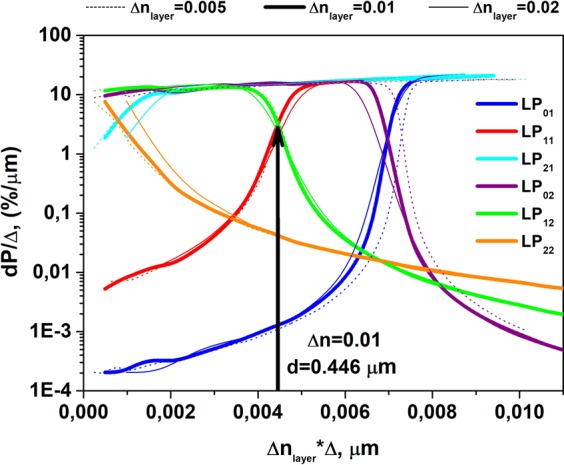
Calculated dependence of the normalized mode’s power fraction located in the high-index ring layer on the optical thickness of the absorbing layer. The dotted, thick solid and thin solid curves correspond to Δn_layer_ equaling 0.005, 0.010, and 0.020, respectively.

Figure 2 shows the fractional powers being localized in the ring layer for the first six groups of modes, LP_i,j_ (i, j = 0,1; 1,1; 2,1; 0,2; 1,2; 2,2), calculated for different values of the layer thickness. The fractional power (y-axis) was normalized to the layer thickness Δ. For the x-axis, we used the value (Δn_layer_ ∙Δ), which will be referred to below as the layer optical thickness. It is interesting that the curves for different Δn_layer_ (in the range 0.005–0.020) coincide in Fig. 2. Referring to Fig. 2, the dP_i,j_/Δ value is an invariant for a fixed absorbing layer optical thickness: the dependences of dP_i,j_/Δ on the optical thickness of the layer coincide for different Δn_layer_.

As mentioned above, the localization of the LP_11_ mode inside the absorbing ring layer results in simultaneous localization of the LP_12_ mode inside the core (see Figs. 1b and 2). The same situation was observed for other pairs of modes: LP_01_ and LP_02_; LP_21_ and LP_22_. In these cases, resonant coupling between each pair of modes occurs at a certain absorbing layer optical thickness where n_eff_ of the both modes become very close one to each other. The coupling results in a disturbance of the mode shape: instead of modes being localized only inside the core or only inside the ring, a “supermode” mixture appear, and significant fractions of each mode become localized in both the core and the absorbing ring. When the absorbing layer optical thickness becomes larger than the “resonance” value, the modes in each pair change their localization (the LP_11_ mode initially localized inside the core becomes localized inside the absorbing ring layer and vice versa for the LP_12_ mode), which is caused by a mode anticrossing effect similar to that discussed in^20^. Nevertheless, when the absorbing ring layer optical thickness is near the resonance value (the optical thickness of the high-index absorbing layer was in this case equal to 0.446μm), both LP_11_ and LP_12_ modes have a noticeable fraction of power inside the high-index absorbing ring layer (see Figs. 1c and 2). In the current work, we chose this design to achieve maximum suppression of the LP_11_ and LP_12_ modes. Variation in the absorbing ring optical thickness within ±5% is still acceptable – more than 1% of the power of each mode localized inside the absorbing ring layer.

**Figure 3:**
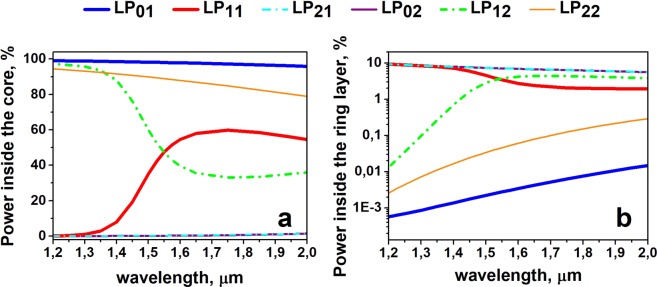
Calculated spectral dependence of the power fraction located in the core (**a**) and high-index absorbing layer of “resonant” width (**b**) for the different modes.

Another important parameter is the wavelength range where efficient suppression of both LP_11_ and LP_12_ modes can be achieved. To clarify this issue, we calculated the dependence of the fractional powers inside the core (see Fig. 3a) and inside the absorbing layer (see Fig. 3b) on the wavelength. The operation spectral range is also quite large, and for wavelengths above 1.4 μm, good LP_11_/LP_12_ mode suppression can be realized.

**Figure 4:**
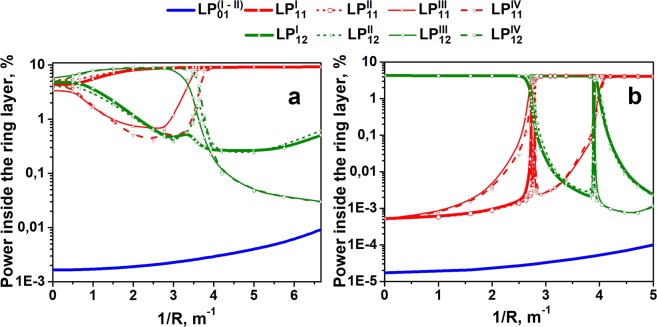
Calculated dependence of the mode fractions inside the absorbing ring layer on the inverse of the bend radius for the fiber structure with core to cladding diameter ratio of 35/80 (**a**) adapted for the case of a straight fiber and 45/125 (**b**) adapted for the case of bending with radius of 0.25 m.

Bend sensitivity is another important parameter in the proposed structure, as it defines the possibility of producing compact fiber lasers by winding active fiber on a spool. The dependence of the fractional powers inside the absorbing layer on the bend radius was calculated for the RIP shown in Fig. 1c. It can be seen (Fig. 4a) that the proposed design is moderately sensitive to bending and HOM suppression exists up to a bending radius of 0.25 m. The observed deterioration of resonance conditions under fiber bending is obviously associated with a change in the effective refractive indexes of the structure modes and resonance conditions, respectively. If a smaller bending radius is required, it will be possible to optimize the absorbing ring layer thickness not for the case of straight fiber but for a fiber coiled on a spool with a given radius.

An important feature of the proposed fiber design is that the single-mode nature of the initial RIP (those with infinite pure silica cladding) no longer plays a critical role. With the RIP, which in the case of an infinite first cladding supports the propagation of a few HOMs, it is possible to suppress the LP_11_/LP_12_ modes in the DC fiber design by insertion of the absorbing layer. To demonstrate this possibility, we optimized the fiber design with a core diameter equals to 45 µm (the RIP shown in Fig. 1a was “stretched” to a larger core diameter). The cladding diameter was 125 µm. The fiber was adapted to operate when wound on a spool with a radius of 0.25 m. For the optimized absorbing layer optical thickness, the dependence of the power fraction inside the absorbing layer on the inverse of the coiling radius of the fiber is shown in Fig. 4b. It can be seen that in some range of bend radii around 0.25 m, it is possible to suppress most of polarization of the unwanted LP_11_/LP_12_ modes. The resonance position where all the polarization states of LP_11_/LP_12_ modes can be efficiently absorbed is very narrow, but we expect that a fast power exchange between polarization states of each group of modes (LP_11_ or LP_12_) will make the “high loss” region to be wide (at least in the range of 1/R from 2.5 to 4.2) – even if one polarization state of the mode has a high optical loss the other one will have high loss too due to power exchange with the “high-loss” polarization state.

### Experiment

**Figure 5:**
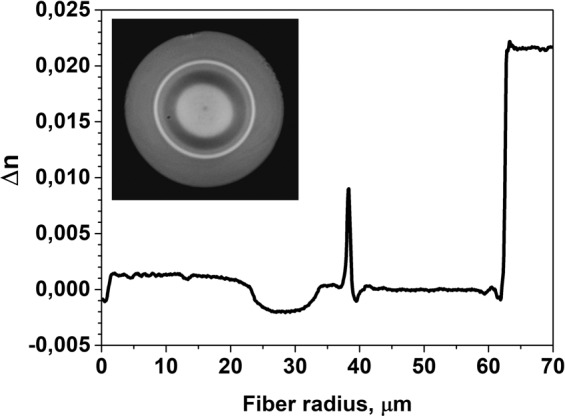
The RIP of the realized fiber measured with an EXFO fiber analyzer NR9200HR (the RI at radii of more than 62.5 μm correspond to the immersion liquid used during measurements); insert: image of the fiber end taken with optical microscope.

To test the proposed conception of HOM suppression, we have realized a 45/125 μm optical fiber with an absorbing layer optimized for operation at a bend radius of 0.25 m (see Fig. 4b). The preform of the fiber layer was fabricated by us using a previously developed “rod-in-tube” method^21^. For this aim, the preform with the W-shaped core RIP used in the modeling (see previous section) was etched in HF to reduce the outer preform diameter down to the designed position of the absorbing layer. Then, for the silica tube with the absorbing layer, a silica layer doped with ~2 mol.% of Al_2_O_3_ and 2 wt.% of Tm^3+^ was deposited on the inner surface of the pure silica glass tube using Modified Chemical Vapor Deposition (MCVD) technique. Then, the tube was jacketed over the etched core preform. The fabricated preform was drawn into a fiber and coated with a low-index acrylate polymer providing NA > 0.45. The measured RIP of the fabricated fiber is shown in Fig. 5.

The mode composition of the fiber near 1.55 μm was investigated by scanning an exciting beam across the fiber diameter and observing the output mode shape with a Spiricon SP-1550M camera. In this case, the shift of the excited beam from to the fiber axis led to excitation of the HOMs and a corresponding output mode shape change. In a perfectly single-mode fiber, only a decrease in the fundamental mode intensity can be observed with this method.

**Figure 6:**
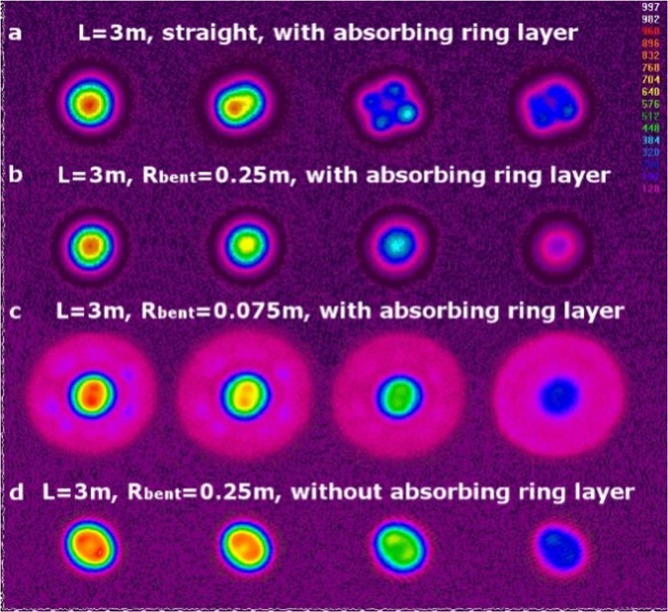
Measured core mode composition of the 45/125 μm fiber with/without an absorbing layer. The images from left to right correspond to monotonic shifts in the excitation beam from the coaxial position to the off-center position.

As shown in Fig. 6b, the realized fiber with a length of 3 m, wound on a spool with a radius of 0.25 m, was perfectly single-mode. The straight fiber with the same length supported propagation of HOMs (Fig. 6a). In the case of the fiber wound on a spool with a smaller radius (7.5 cm), the observed cladding intensity (see Fig. 6c) resulted from coupling of the LP_01_ mode with a ring mode due to bending. The investigation of the optical fiber with an identical RIP (drawn from the same core preform) but without an absorbing ring layer demonstrated the presence of the LP_11_ mode in the 3 m sample bent with a radius of 0.25 m (see Fig. 6d).

## Fiber with high-index rods

### Structure design

The modeling and experiment described in the previous section proved the possibility of HOMs suppressing by introducing an absorbing ring layer into the first cladding of the fiber. However, only one group of HOMs (pairs of LP_11_/LP_12_ modes in the above-mentioned case) can be efficiently suppressed using one ring layer, when other high-order modes have low propagation loss (see, for example, mode LP_22_ in Figs. 2 and 3).

The next part of our investigation is devoted to the suppression of unwanted HOMs using absorbing rods inserted into the first fiber cladding. An important advantage of this approach is the possibility of inserting several absorbing rods, each designed for the suppression of specific groups of HOMs. In addition, the design with absorbing rods is simpler and more reliable in terms of fiber preform fabrication technology. Indeed, both the core and rods preforms can be fabricated separately, and the diameters can be optimized and controlled with a high accuracy before consolidating the core and absorbing rods together.

Similar to the previous section, for the calculations we used a preform with a W-shaped core RIP available in stock. The core diameter of the designed fiber was chosen to be 32 μm, the diameter of the cladding was 120 μm, the Δn_core_ was 0.0018, and the core was surrounded by a depressed layer with Δn_depr_ = −0.0022. According to the calculations, several modes were localized in the core of the fiber: the fundamental mode LP_01_ and the LP_11_, LP_21_, LP_02_ modes. It is worth noting that the LP_02_ mode was under the cut-off wavelength for the optical fiber with an infinite first cladding, but due to the presence of the secondary reflecting cladding, its power was mainly localized in the core (~80%).

**Figure 7:**
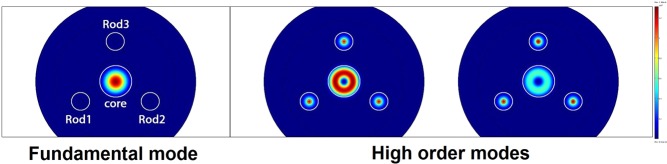
Calculated 2D mode field intensity distribution of the fundamental mode (left) and first two high order modes.

In the current work, we chose an optical fiber design with three absorbing rods located equidistant from the fiber axis, forming an equilateral triangle. This spatial configuration provides maximum distortion of HOMs^22^. In our design, a specific HOMs were evacuated from the fiber core due to resonant coupling with fundamental mode of the high-index absorbing rods. Figure 7 shows the calculated two-dimensional intensity distribution of the fundamental mode LP_01_ and two first high-order modes. It can be seen that high order modes are noticeably distorted by the rods introduction and will be suppressed if rods have a high absorption.

**Figure 8:**
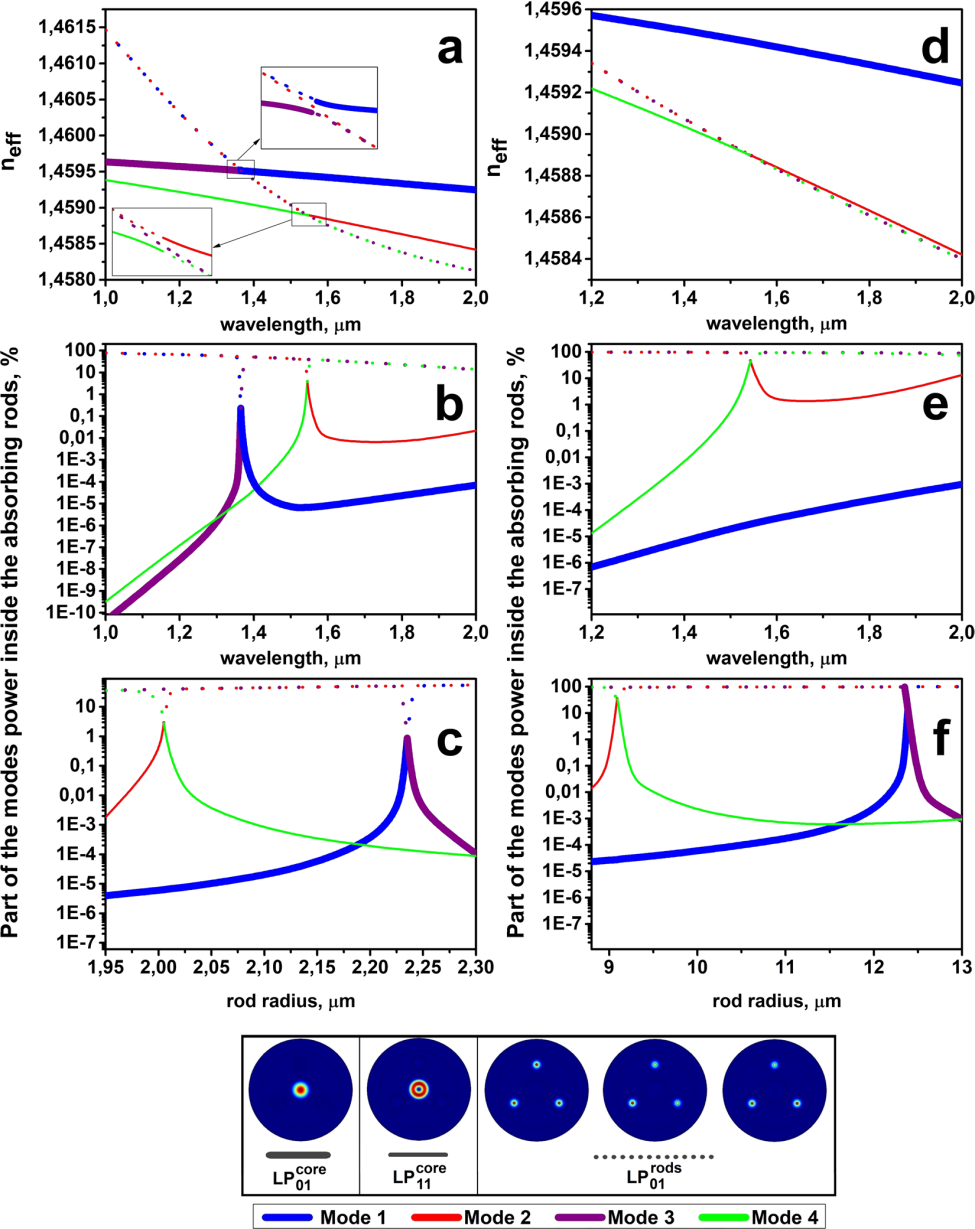
(**a,d**) Calculated wavelength dependence of the effective indexes n_eff_ for the modes propagating in the designed waveguide structure; (**b,e**) dependence of the modes fractional powers inside the absorption rods; (**c,f**) dependence of the modes fractional powers in the absorption rods on rod radius. Parts (**a**–**c**) correspond to Δn_rods_ = 0.009; parts (**d**–**f)** correspond to Δn_rods_ = 0.0013. Line of one color designates eigen mode of the structure. Line type (bold line, thin line and dots) define mode’s localization.

It must be noted that in fiber with few waveguide elements (in our case with core and three rods) the fundamental mode (mode with the highest effective index) might be located in the cladding high-index inclusions and the mode, which we assume to be fundamental (which is located in the central core and has intensity distribution corresponding to the fundamental mode) can be one of HOMs of such a system. To simplify understanding we designated eigen modes of such a system just as “Mode 1”, “Mode 2” and etc. For the certain wavelength the mode, which is preferable located in the central core we will call core mode LP^core^_i,j_, assuming that such mode has parameters (intensity distribution and effective index) similar to the LP_i,j_ mode of the separate core (without high index rods). The modes, located in the high-index rods we will call the absorbing rod mode LP^rods^_i,j_ using the same principle. Near the anticrossing resonance between the core mode and absorbing rod mode localization of eigen modes is changing and fundamental core mode (LP^core^_01_), which was mode #1 at some wavelength, become mode #3 for another wavelength (see Fig. 8). To make this feature visible at the figures we mark each eigen mode (#1, #2 and etc) by separate color. The difference between core and absorbing rods modes is shown by line type (bold line is the fundamental core mode, thin lines are LP^core^_11_ mode and dots shows the modes localized in absorbing rods).

The resonance can be easily observed for a fixed wavelength when the absorbing rod parameters (size and refractive index) are properly adjusted. However, in practice, the accuracy of fiber profile fabrication relative to designed one is important, and we studied its influence on the fiber characteristics. A design of the fiber with three identical rods was considered. The size of the rods was chosen to provide resonant coupling at the operating wavelength of 1.55 μm between polarization components of the LP^core^_11_ mode and LP^rods^_01_ modes. First, we consider the case in which the refractive index difference between rods and pure silica cladding Δn_rods_ was several times larger than the core-cladding refractive index difference Δn_core_ (Fig. 8a–c). For the optimal fiber design, a sufficient localization of the LP^core^_11_ mode power inside the absorbing rods (in excess of 1%) can be observed in a very narrow spectral region of approximately 0.01 μm (Fig. 8b). The region where the loss of the LP^core^_11_ mode exceeds that of the fundamental core mode LP^core^_01_ by at least 1000 times is somewhat wider (up to 0.07 μm), but a relatively long fiber length might be required in this case to achieve noticeable attenuation of the LP^core^_11_ mode. It is also interesting to note that around 1.37 μm not HOM of the core, but the fundamental mode of the core LP^core^_01_ had resonant coupling with absorbing rods fundamental modes LP^rods^_01_.

It is worth noting that a design with rods having a high refractive index is rather sensitive to the rods diameter (in the assumption that the refractive index is fixed). A change in the rod diameter from the design by only 12% (Fig. 8c) will lead to a delocalization of the fundamental core mode LP^core^_01_. To match the resonance for HOM core mode LP^core^_11_ (keeping the mode’s power inside the absorbing rods to be more than 0.1%), the tolerance of rod diameter should be smaller than 1%. Thus, from a practical point of view, the structure with a high Δn_rods_ has a small practical interest.

Next, we examined a fiber design with Δn_rods_ close to the Δn_core_ (Fig. 8d–f). Additionally, to minimize the penetration of the fundamental core mode LP^core^_01_ into the region of the absorbing rods, we chose the profile of the rods with a depressed layer. As shown in Fig. 8d,e, phase matching between the first HOM of the core LP^core^_11_ and the rod’s mode LP^rods^_01_ can be achieved in a very wide spectral range in this case. Approximately 1% of the first core HOM LP^core^_11_ is localized in the absorbing rods for wavelengths from 1.5 μm to above 1.9 μm. The difference in attenuation between the fundamental core mode LP^core^_01_ and the first HOM core mode LP^core^_11_ exceeds 1000 times over this spectral range. Additionally, the requirements for the tolerance of the absorbing rod diameter (Fig. 8f) become slightly more realistic. The loss ratio of the core fundamental mode (Mode 1) and the first HOM (Mode 2) is more than 3 orders of magnitude when the fiber preform manufacturing accuracy is less than 2%. Resonant suppression of the fundamental core mode LP^core^_01_ occurs only when the diameter of the rods deviates from the optimal value by approximately 40%. This design was chosen for further analysis.

**Figure 9:**
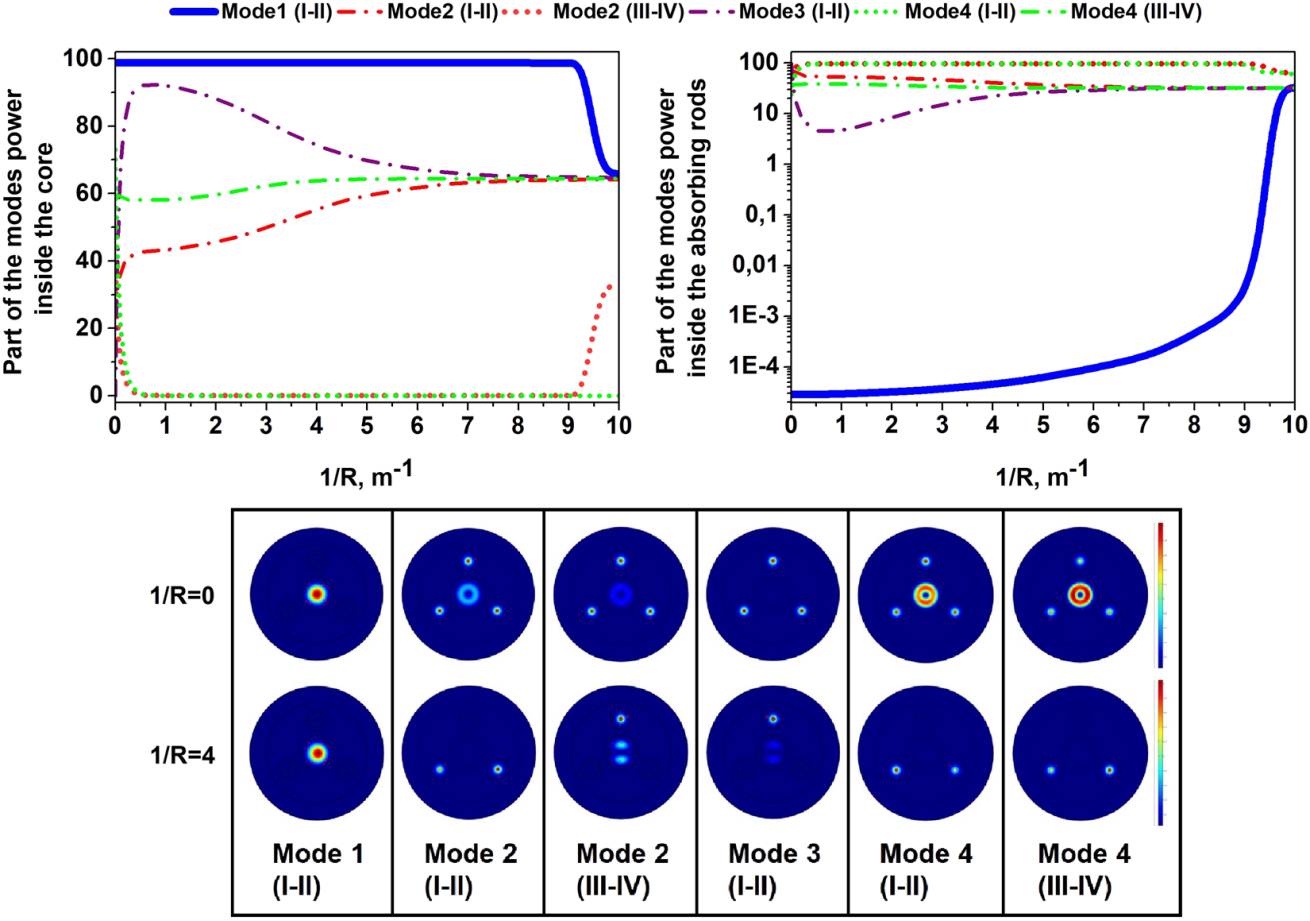
Calculated dependence of the Mode 1, Mode 2, Mode 3, Mode 4 fractional powers inside the absorbing rods on the bending radius. The index number of polarization components is shown in Roman numerals in brackets.

To analyze the bend sensitivity of the designed structure, we calculated the dependence of the fraction of the mode’s power inside the absorbing rods on the bending radius. The results for different polarization states of the Mode 1 (LP^core^_01_ mode), Mode 2, Mode 3, Mode 4 groups (hybrid modes appeared due to resonant coupling between LP^core^_11_ modes and LP^rods^_01_ modes) averaged for different bending directions are shown in Fig. 9. When the bending radius exceeds 11 cm, the ratio between the losses of the fundamental core mode and first HOM of the core is still more than 1000 times. So the design is relatively insensitive to bending.

### Experiment

As mentioned above, in the case of our core design there are a few groups of HOMs localized inside the core. By the next step we have modify fiber design by adding absorbing rods of different size. Two absorbing rods were optimized to achieve resonance with the first core HOM (LP^core^_11_ mode). In the calculations, the use of two rods simultaneously was necessary for efficient suppression of all polarization states of the first HOM (LP^core^_11_ mode). A third rod was added to suppress the LP^core^_21_ mode.

The fiber fabrication process consisted of several steps. First, the diameter of the MCVD passive fiber preform available from a stock was adjusted (by etching/jacketing) to match the designed core/clad ratio. Then, three holes were drilled in the position where absorbing rods should be placed. The absorbing rod preform was also fabricated by the MCVD process. The core of the absorption rods was based on a phosphoroaluminosilicate glass matrix, which allows simultaneous achievement of high rare-earth element concentrations and low numerical apertures^23^. Similar to the previous section, Tm^3+^ was chosen as an absorbing element (the concentration of thulium was approximately 1.2 wt%). The absorbing rod preform was etched and stretched to match the drilled holes and the final fiber design. The fiber preform and absorbing rods were consolidated together and drawn into fibers with different outer diameters. This process was technically identical to that used for production of polarization-maintaining “PANDA”-type fibers, so no defects or imperfections were observed near the absorbing rods. The fibers were coated with a low-index polymer providing a NA > 0.45.

**Figure 10:**
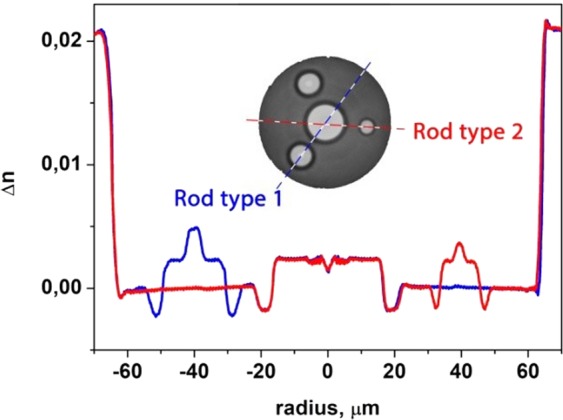
Measured fiber RIPs. The colored lines show the measured RIP cross-section, and the inset shows an image of the fiber end obtained with an optical microscope.

The RIPs of the realized fibers, measured in different cross-sections (passing through the fiber axis and one of the rods), are shown in Fig. 10. As in the case of the fiber with absorbing ring layer refractive index profile of the core was W-shaped^17,18^. The inset shows an image of the fiber end. It is important to note that due to the non optimal drawing conditions (high tension of the fiber in the drawing process), the refractive index of silica glass in the fiber decreased by approximately 0.001^24^. As a result, the Δn of the rods and the core relative to silica increased. Thus, the effective indexes (propagation constants) of the modes changed as well as the resonance conditions for mode coupling.

**Figure 11:**
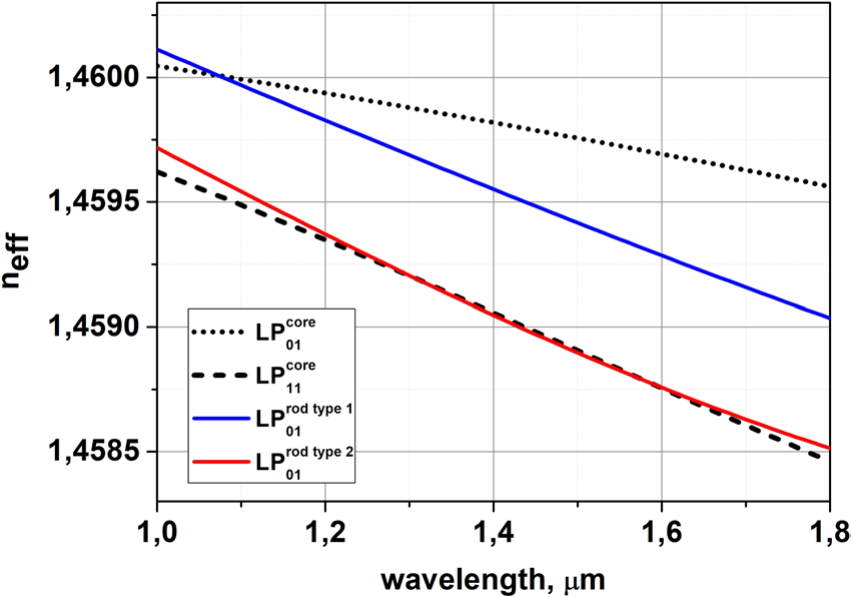
Calculated n_eff_ for modes of the realized fiber with a cladding diameter of 90 μm.

Mode composition analysis showed that the suppression of core HOMs can be observed in the fiber with an outer cladding diameter of 90 μm. Both the core and the absorbing rods has non-step-index refractive index profile, so modes analysis with COMSOL become cumbersome. To simplify analysis we calculate modes propagated in a separate core (without high index rods) and in a separate absorbing rod. We have found that detached core guides two modes at 1.55 µm: fundamental mode and first HOM. Calculation of n_eff_ dependence for these modes on wavelength is shown in Fig. 11. Also we plot at this graph the n_eff_ calculated for the fundamental mode of detached absorbing rod. It can be seen that in the 1.55 μm spectral region, n_eff_ of the first HOM matches n_eff_ of the fundamental mode of detached high-index rod of type 2, so resonant coupling between these modes can be expected in the fabricated fiber.

**Figure 12:**
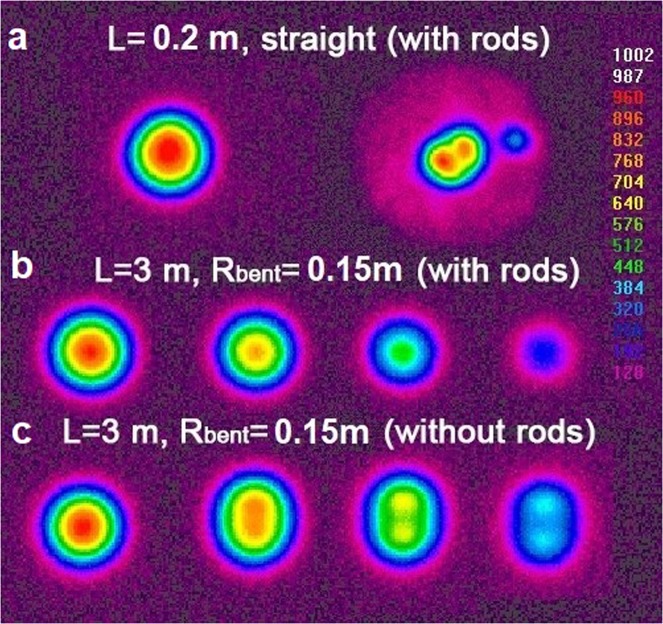
Mode composition measured on the output end of the fiber with outer cladding diameter of 90 μm.

As before, we investigated the mode composition of the fiber by scanning an exciting beam across the fiber diameter (see Fig. 12). In the first experiment, we investigated a short length (L = 0.20 m) of the straight fiber. The fundamental mode (LP^core^_01_) and the first HOM (LP^core^_11_) were observed by coaxial and off-center excitation, respectively (see Fig. 12). It is worth noting that with the excitation of the first HOM, we observed intensity in the region of the smallest absorbing rod, which confirms the resonant coupling between the first HOM (LP^core^_11_) and the absorbing rod mode (LP^rods^_01_). The direct excitation of the core fundamental mode did not lead to a similar intensity for the absorbing rods. The bending of the fiber increased the coupling between the first HOM and the resonant mode. In this case, it was possible to eliminate the HOMs from the fiber core when a 3-meter-long fiber was wound on a spool with a radius of 0.15 m (Fig. 12). The same asymptotically single-mode propagation regimes were observed for the cases of fibers bent with radii down to 0.07 m. A further decrease in the bending radius was accompanied by fundamental mode leakage into the cladding: the excitation of the core modes led to increase of light intensity in the fiber cladding. It is necessary to note that a similar fiber without absorbing rods (drawn in the same conditions from the same core preform) under identical bending conditions (R_bend_ = 0.15 m) supported propagation of both the fundamental mode and the first HOM (see Fig. 12). Thus, the possibility of the first HOM suppression with the help of absorbing rods was experimentally confirmed.

The absorption inside the rods was estimated to be approximately 60 dB/m at 1.55μm. Core losses were experimentally measured by the cutback method. In this case, the initial (30 m) and cutback (3 m) length of the fiber were wound on a spool with a diameter of 0.30 m (to guarantee propagation in the core only via the fundamental mode). The loss of the fundamental mode at a wavelength of 1.55 μm did not exceed 0.2 dB/m. The level of propagation loss measured as absorption from the cladding at the pump wavelength (0.98 μm) did not exceed 0.1 dB/m.

## Discussion and conclusions

In the present paper, we proposed a new approach for the suppression of undesirable modes in DC optical fibers with an increased core-to-cladding diameter ratio. The approach is based on the introduction of high-index absorbing inclusions (ring layer or rods) into the first fiber cladding. Properly adjusted inclusion parameters disturbed the undesirable modes shape and increased their propagation losses due to absorption of the power located inside the inclusions. In the experiments, we tested two fiber designs: a cylindrically symmetric design (the core was surrounded with an absorbing ring layer) and a design with absorbing rods. In both cases, single-mode core propagation was demonstrated, confirming the efficiency of the proposed approach.

It is worth noting that in accordance with the experimental results, the loss of the unwanted modes was significantly higher than that predicted by the calculations. In the case of the fiber with absorbing rods, due to fabrication errors, only one small rod (type 2) allowed resonant coupling with the first HOM. Nevertheless, the suppression of the unwanted HOMs was very efficient in the bent fiber. It can be suggested that in reality, there is an additional mechanism that enhances the HOM suppression. It can be associated with energy transfer between modes with close n_eff_ due to perturbation of fiber, i.e. bending and twisting. For example, previously strong resonant coupling was observed in Bragg fibers^20^, few-mode hybrid fibers^25,26^, and due to twist in the so-called GT-Wave fiber bundles^27^, such a condition led to the modes power part redistribution. In our case there is the interaction of polarization states of one group of modes. If one polarization state has high optical loss, the other will be efficiently attenuated due to energy exchange between the modes of one group (with the assumption of close n_eff_). Also energy transfer occurred between the unwanted core HOM and the absorbing rods mode. Near the resonance, a small difference in n_eff_ can also enhance mode power exchange in this case and in this way increase the attenuation of the HOMs (the mode located inside the absorbing rod has a very high attenuation). Thus, due to energy exchange with the absorbing rod mode, unwanted core HOMs can be efficiently suppressed. In this situation, the tolerance of the absorbing inclusion parameters becomes much less critical. Indeed, to achieve efficient power transfer between modes on resonance without very strong delocalization of the undesirable mode, power in the range of 0.1–1% located inside the absorbing rod seems to be sufficient, with fast energy exchange between the core and rod fiber.

It is worth to note that the designed structured with absorbing rods looks similar to the Chirally-Coupled Core (3C) fibers^28,29^, where high order modes evacuated from the core due to coupling with side core(s) modes. In 3C fibers the side core is highly bent (it rotates around the main core with a very short period), so modes located in it have a high bend loss. In our case application of absorbing inclusions makes fiber structure to be much more flexible - circular symmetric as well as polarization maintaining structures with efficient suppression of HOM scan be designed. Moreover, fiber design with absorbing inclusion is much simpler in production as no modification of the drawing process is required.

## Methods

To calculate mode’s power fraction and distributions in the case of the cylindrically symmetric fiber design, we used in-house built software for numerical solving of scalar wave equation and the COMSOL software package for the calculation of mode field intensity distribution in the bent fiber structures and in the structure with three absorbing rods. The mode composition of the fiber near 1.55 μm was investigated by scanning an exciting beam across the fiber diameter and visual observing the output mode shape with a Spiricon SP-1550M camera. Core losses were experimentally measured by the cutback method.
